# Uncommon association: *Pseudomonas luteola* bacteremia in an immunocompetent individual with acute tonsillitis – A case report

**DOI:** 10.1016/j.idcr.2023.e01891

**Published:** 2023-09-04

**Authors:** Sirine Ahmad, Ahmad J. Alzahrani, Mohammed Alsaeed

**Affiliations:** a Medicine Department, Dr. Sulaiman Al Habib Medical Group, Riyadh, Saudi Arabia; b College of Medicine, Al-Imam Mohammed Ibn Saud Islamic University, Riyadh, Saudi Arabia; c Laboratory Department, Dr. Sulaiman Al Habib Medical Group, Riyadh, Saudi Arabia; d Medicine Department, Infectious Diseases Division, Prince Sultan Military Medical City, Riyadh, Saudi Arabia; e Alfaisal University, College of Medicine, Riyadh, Saudi Arabia

**Keywords:** *Pseudomonas luteola*, Bacteremia, Acute tonsillitis

## Abstract

*Pseudomonas luteola*, formerly known as *Chryseomonas luteola*, is an infrequently encountered aerobic gram-negative bacterium. While it has been identified as a potential human bacterial pathogen, its connection to specific clinical conditions remains limited. Here, we present an exceptional case of a 27-year-old immunocompetent man with acute tonsillitis, who developed *P. luteola* bacteremia. This unique correlation, not extensively documented in previous studies, sheds light on the potential pathogenicity of *P. luteola* in patients with acute tonsillitis.

## Introduction

*Pseudomonas luteola*, characterized by its distinctive yellow pigment, is a gram-negative, motile, and strictly aerobic rod. Differentiating from other yellow-pigmented non-fermenters due to its negative oxidase reaction, *P. luteola* is primarily found in environments with high moisture content [1], [2]. Although infrequently reported in the literature, *P. luteola* bacteremia has mostly been associated with immunocompromised individuals and those with indwelling catheters. We present a case of *P. luteola* bacteremia in an immunocompetent patient with acute tonsillitis, a unique occurrence not previously extensively studied [3].

## Case report

A 27-year-old man with no known comorbidities; sexually active with single female partner and no history of illicit drug use or animal contact. Arrives to the emergency department (ED) complaining of fever and severe sore throat for three days. Notably, three years prior, he experienced frequent episodes of tonsillitis. The patient was hemodynamically stable at presentation and was discharged while on oral amoxicillin.

However, two days later, the patient experienced worsening symptoms. He returned to the ED and presented with the following vital signs: body temperature, 39 °C; blood pressure, 110/90 mmHg; and pulse rate, 125 beats per minute. Physical assessment revealed the presence of bilateral enlargement of the lymph nodes with erythematous and enlarged tonsils. Furthermore, he had a white blood cell count of 17,000/microliter, neutrophil count of 88%, C-reactive protein (CRP) level of 192 mg/L, erythrocyte sedimentation rate of 61 mm/h and HIV screening was negative. The chest radiograph showed normal findings. Additionally, one set of blood culture was collected on admission.

The patient was admitted to the hospital with severe tonsillitis and treated with 600 mg of clindamycin orally every 8 h. After 24 h, the patient remained febrile 38.4 ^o^C and complained of severe neck pain. Computed tomography of the neck revealed nasopharyngeal adenoid and tonsillar hypertrophy, as well as multiple bilateral enlarged cervical lymph nodes. The patient’s fever persisted until the third day of hospitalization. The CRP levels increased to 256 mg/L.

**Fig. 1:**
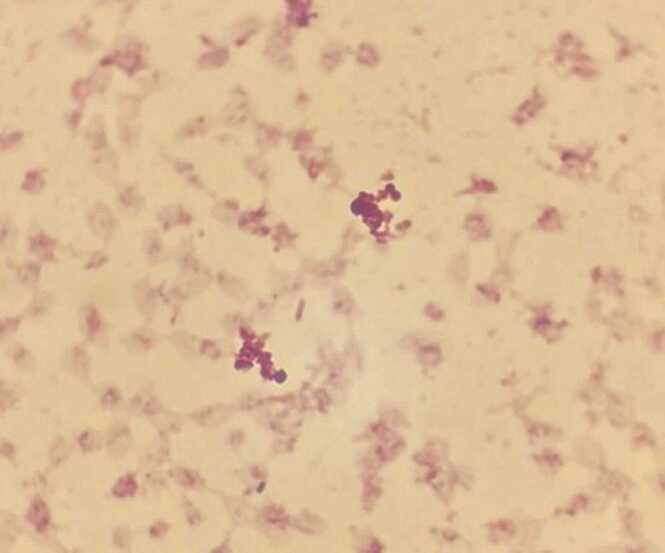
Gram stain revealed gram-negative bacilli.

**Fig. 2:**
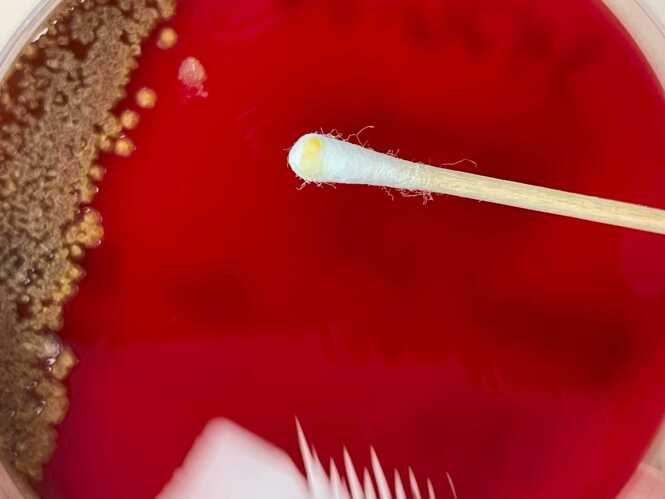
Yellow-colored colonies appeared on Blood agar after 24 h.

The aerobic and anaerobic bottles were fitted in the BACT/ALERT 3D blood culture system (Biomérieux, Durham, NC, USA). After 29 h, the aerobic bottle turned positive, and the gram stain revealed gram-negative bacilli (Fig. 1). Clindamycin was discontinued, and meropenem was initiated. 24 h later, yellow-colored colonies appeared on blood agar after 24 h (Fig. 2), non-lactose fermenter on Macconkey agar plate. The oxidase test was negative. Using the GN card Vitek 2 (Biomérieux, Inc., Hazelwood, MO), the strain was identified as *P. luteola*. The antibiotic susceptibility of this organism was examined using the E-test gradient MIC (Biomérieux, France). It demonstrated susceptibility to amikacin, ceftazidime, ciprofloxacin, piperacillin/tazobactam, meropenem and trimethoprim/sulfamethoxazole.

The patient’s fever subsided 24 h after stating meropenem, and inflammatory biomarker levels began to decline. His condition stabilized, and he was eventually discharged with a 10-day course of ciprofloxacin. Two weeks after discharge, outpatient follow-up showed complete recovery and a plan for elective tonsillectomy with head and neck surgery.

## Discussion

*P. luteola*, previously classified as a CDC group Ve-1, is a gram-negative, aerobic, non-spore-forming, motile, non-lactose fermenting, and oxidase-negative bacillus. This organism grows on MacConkey agar and produces yellow-pigmented colonies [1], [2].

*P. luteola* is a relatively rare opportunistic pathogen, commonly observed in their saprophytic form in various environments such as soil, water, and other areas with high moisture content. Moreover, they exhibit opportunistic pathogenicity in individuals who have preexisting medical conditions or have indwelling catheters [3].

The use of steroids and other immunosuppressive medications, the presence of a foreign body, and surgical instability predispose patients to develop *P. luteola* infection. Therefore, nosocomial infections are more common than community-acquired infections [3].

The documented cases encompass a range of conditions such as bacteremia [4], [5], empyema [6], endocarditis [7], peritonitis [8], and meningitis [9]. The number of healthy patients who develop these conditions is relatively few [10], [11]. To the best of our knowledge, this case report is the first to document the occurrence of *P. Luteola* bacteremia associated with acute tonsillitis in an immunocompetent patient.

A previous retrospective study by Bayhan et al., conducted in a Turkish tertiary care facility, reported an incidence of *P. luteola* bacteremia in seven pediatric patients over the course of a nine-year period of study. All have congenital diseases or primary immunodeficiencies. Among these patients, the infection was hospital acquired in six and community acquired in one [3].

According to some previous studies, *P. luteola* exhibits high resistance to trimethoprim-sulfamethoxazole, ceftriaxone, tetracycline, and ampicillin [11], and demonstrates susceptibility to other antibiotics, including ceftazidime, meropenem, aminoglycosides, and ciprofloxacin.

A study was previously conducted on febrile patients with cancer admitted to a referral hospital in Ethiopia to identify unusual bacterial pathogens that cause bloodstream infections, and their medication resistance profiles [12]. Of the 107 adult patients with cancer having fever, 13 (12.2%) had uncommon human infections. *P. luteola* was isolated from two neutropenic individuals with non-Hodgkin lymphoma. Both isolates were resistant to amoxicillin-clavulanic acid, ampicillin, chloramphenicol, ceftriaxone, and trimethoprim-sulfamethoxazole.

However, the study by Bayhan showed that all *P. luteola* strains were susceptible to amikacin, gentamicin, trimethoprim-sulfamethoxazole, and meropenem, but resistant to piperacillin-tazobactam, aztreonam, and colistin [3]. Therefore, the *P. luteola* isolates display different levels of susceptibility to antibiotics.

## Conclusion

This unprecedented case underscores the potential for *P. luteola* to cause bacteremia in patients with acute tonsillitis, even in immunocompetent individuals. The rarity of such occurrences necessitates further research into the underlying mechanisms, treatment strategies, and potential impacts on healthcare systems and patients. This case report serves as a reminder that unusual pathogens can surface in unexpected clinical contexts, prompting a need for ongoing vigilance and research.

## Funding

The authors received no specific grant from any funding agency.

## Ethical approval

All authors have agreed to authorship, read and approved the manuscript, and given consent for publication of the manuscript.

## Consent

Consent to publish was not obtained since the case report does not contain any personal identifiers.

## CRediT authorship contribution statement

Sirine Ahmad: Drafted first version of the manuscript and medical care of the patient. Ahmad J. Alzahrani: Laboratory diagnosis and writing the microbiology section. Mohammed Alsaeed: Provided feedback on manuscript and medical care of patient.

## Declaration of Competing Interest

All authors report no potential conflicts of interest.
